# Factors influencing time to diagnosis and initiation of treatment of endemic Burkitt Lymphoma among children in Uganda and western Kenya: a cross-sectional survey

**DOI:** 10.1186/1750-9378-8-36

**Published:** 2013-09-30

**Authors:** Geoffrey C Buckle, Jennifer Pfau Collins, Peter Odada Sumba, Beccy Nakalema, Dorine Omenah, Kristine Stiffler, Corey Casper, Juliana A Otieno, Jackson Orem, Ann M Moormann

**Affiliations:** 1 University of Massachusetts Medical School, 55 Lake Avenue North, Worcester 01655, MA, USA; 2 School of Medicine, Case Western Reserve University, 10900 Euclid Ave, Cleveland 44106, OH, USA; 3 Center for Global Health Research, Kenya Medical Research Institute, Kisumu 40100, Kenya; 4 Uganda Cancer Institute, Upper Mulago Road, Kampala, Uganda; 5 Fred Hutchinson Cancer Research Center, 1100 Fairview Ave N., Seattle 98109, WA, USA; 6 Jaramogi Oginga Odinga Teaching and Referral Hospital, Kisumu 40100, Kenya; 7 Department of Pediatrics, University of Massachusetts Medical School, 373 Plantation Street, Suite 318, Worcester 01605, MA, USA

**Keywords:** Africa, Kenya, Uganda, Cancer, Children, Burkitt lymphoma, Delay, Delayed diagnosis

## Abstract

**Background:**

Survival rates for children diagnosed with Burkitt lymphoma (BL) in Africa are far below those achieved in developed countries. Late stage of presentation contributes to poor prognosis, therefore this study investigated factors leading to delays in BL diagnosis and treatment of children in Uganda and western Kenya.

**Methods:**

Guardians of children diagnosed with BL were interviewed at the Jaramogi Oginga Odinga Teaching and Referral Hospital (JTRH) and Uganda Cancer Institute (UCI) from Jan-Dec 2010. Information on sociodemographics, knowledge, attitudes, illness perceptions, health-seeking behaviors and prior health encounters was collected using a standardized, pre-tested questionnaire.

**Results:**

Eighty-two guardians were interviewed (20 JTRH, 62 UCI). Median "total delay" (1st symptoms to BL diagnosis) was 12.1 weeks [interquartile range (IQR) 4.9-19.9] in Kenya and 12.9 weeks (IQR 4.3-25.7) in Uganda. In Kenya, median "guardian delay" (1st symptoms to 1st health encounter) and "health system delay" (1st health encounter to BL diagnosis) were 9.0 weeks (IQR 3.6-15.7) and 2.0 weeks (IQR 1.6-5.8), respectively. Data on guardian and health system delay in Uganda were only available for those with < 4 prior health encounters (n = 26). Of these, median guardian delay was 4.3 weeks (range 0.7-149.9), health system delay 2.6 weeks (range 0.1-16.0), and total delay 10.7 weeks (range 1.7-154.3). Guardians in Uganda reported more health encounters than those in Kenya (median 5, range 3–16 vs. median 3, range 2–6). Among Kenyan guardians, source of income was the only independent predictor of delay, whereas in Uganda, guardian delay was influenced by guardians’ beliefs on the curability of cancer, health system delay, by guardians’ perceptions of cancer as a contagious disease, and total delay, by the number of children in the household and guardians’ role as caretaker. Qualitative findings suggest financial costs, transportation, and other household responsibilities were major barriers to care.

**Conclusions:**

Delays from symptom onset to BL treatment were considerable given the rapid growth rate of this cancer, with guardian delay constituting the majority of total delay in both settings. Future interventions should aim to reduce structural barriers to care and increase awareness of BL in particular and cancer in general within the community, as well as among health professionals.

## Background

Endemic Burkitt Lymphoma (BL) is the most common childhood cancer in equatorial Africa and in other regions with holoendemic malaria [1,2]. In these areas, BL represents more than half of all childhood cancers and up to 90% of all lymphomas [3]. A characteristic feature of BL is its rapid doubling time (24 to 48 hours) and high proliferation index (near 100%), which makes BL highly susceptible to chemotherapy [4]. In high-income (HIC) countries, where appropriate treatment and supportive care are typically available, cure rates for BL exceed 90% [5,6]. However, survival rates for children with BL receiving treatment in many parts of Africa remain far lower. Most recent studies on the optimal management of BL in this setting have reported cure rates ranging from 48 to 67% [7,8].

In equatorial Africa and other low-income settings, poor outcomes of childhood cancers can be attributed to several factors, including abandonment of therapy, scarcity of medication and supplies for comprehensive care, nutritional deficiencies, inaccurate diagnostics, and treatment-related mortality. Another key cause of treatment failure is late stage of presentation, underscoring the importance of prompt diagnosis and early treatment. Among children with BL, advanced stage has been shown to be associated with poorer response to first-line therapy [9]. With few second-line therapeutic options, late stage of presentation is therefore believed to be a principal barrier to improving long-term survival among children with BL in these settings [10].

Reasons for delayed diagnosis of childhood cancers in sub-Saharan Africa have been previously unexplored. The objective of our study was to investigate the health-seeking experiences of guardians of children specific to BL because of its evident physical manifestations and to identify factors that may lead to delays in these settings. Results from this study may also be applicable to other childhood cancers in Uganda and western Kenya.

## Results

### Sociodemographic characteristics of the guardians

A total of 82 guardians of children diagnosed with BL were enrolled in our study: 62 at Uganda Cancer Institute (UCI) and 20 at Jaramogi Oginga Odinga Teaching and Referral Hospital (JTRH), (formerly the New Nyanza Provincial General Hospital) in Kisumu, Kenya. Table 1 summarizes the sociodemographic characteristics of participating guardians. In brief, the majority of respondents were mothers of the child diagnosed with BL (Kenya 80%; Uganda 50%) and self-identified as primary caretaker (Kenya 90%, Uganda 93%). Half or more worked as peasant farmers (Kenya 50%, Uganda 68%) and the majority reported prior education at primary level or below (Kenya 70%, Uganda 76%). The mean age of the guardian was 36 (range 22–60) and 39 (range 22–65) years in Kenya and Uganda, respectively. There were no significant differences in guardians’ sociodemographic characteristics between the two sites.

**Table 1:** Demographic and socio-economic characteristics of the guardians of children with Burkitt lymphoma at Jaramogi Oginga Odinga Teaching and Referral Hospital (JTRH) and Uganda Cancer Institute (UCI), 2010

	**Western Kenya, n (%)**	**Uganda, n (%)**
**Total**	20 (100.0)	62 (100.0)
Guardian		
Mother	16 (80.0)	31 (50.0)
Father	2 (10.0)	12 (19.4)
Grandparent	0 (0)	12 (19.4)
Aunt	1 (5.0)	3 (4.8)
Sibling	1 (5.0)	3 (4.8)
Self	0 (0)	1 (1.6)
Age (years)	Mean 36.3 (range 22–60)	Mean 39.3 (range 22–65)^a^
Primary Caretaker		
Yes	18 (90.0)	56 (90.3)
No	2 (10.0)	6 (9.7)
Number of children in household		
1-2	4 (20.0)	12 (20.0)
3-4	8 (40.0)	20 (33.3)
≥ 5	8 (40.0)	28 (45.2)
Education		
Did not complete primary education	8 (40.0)	38 (61.3)
Completed primary education	6 (30.0)	9 (14.5)
Attended secondary education	1 (5)	6 (9.7)
Completed secondary education	5 (25)	4 (6.5)
Other	0 (0)	5 (8.1)
Occupation		
Peasant farmer	10 (50.0)	42 (67.7)
Self-employed/Small business	6 (30.0)	8 (12.9)
Other	4 (20.0)	12 (19.4)
Religion		
Christianity	20 (100.0)	52 (83.9)
Pentecostal	10 (50.0)	13 (21.0)
Catholic	4 (20.0)	22 (35.5)
Anglican	3 (15.0)	13 (21.0)
Seventh Day Adventist	1 (5.0)	4 (6.5)
Other	2 (10.0)	0 (0)
Islam	0 (0)	1 (1.6)
None	0 (0)	9 (14.5)
Language of the Interview	Duoluo – 15 (75.0)	Lugandan – 55 (88.7)
	Swahili – 3 (15.0)	Multiple – 2 (3.2)
	English – 2 (10.0)	English – 5 (8.1)

^a^9 missing or unknown.

### Onset of child’s illness: symptoms and guardian’s perceptions

Guardians in Kenya and Uganda recounted noticing similar symptoms at the onset of illness, which were largely non-specific in their presentation. More than half of respondents in both settings said painless swelling of the neck, face, chest, abdomen, underarm or groin was present when the child’s illness was first identified. Kenyan children also commonly had experienced fever, fatigue, weight loss or night sweats (80%), whereas bleeding gums or loose of painful teeth were commonly observed among children in Uganda (31%) with mandibular lesions.

Although early symptoms were mostly similar in both settings, guardians’ initial perceptions of the illness varied between the two sites. Twenty-six of the 62 guardians (42%) in Uganda had suspected the illness was a dental problem or cyst, 12 (19%) believed their child to have a tumor, and 7 (11%) attributed the illness to an injury. Notably, 3 guardians (5%) thought it was a curse or witchcraft. Of the 20 guardians in Kenya, 7 (35%) initially perceived the illness to be splenomegaly or abdominal swelling (referred to as 'hima’ in the Kenyan Luo language) and 6 (30%) malaria. No Kenyan guardian initially suspected cancer nor attributed their child’s illness to a curse or witchcraft.

### Length of delay

The median total delay as reported by guardians of children with BL was 12.1 weeks (range 2.0-308.6) in western Kenya and 12.9 weeks (range 1.7-154.3) in Uganda (mean 38.3 weeks standard deviation 77.3, and 20.4 weeks standard deviation 25.6, respectively). In Kenya, the median guardian delay was greater than the median health system delay at 9.0 (range 0–298.0) and 2.0 weeks (range 0.6-21.4) respectively (mean 34.0 weeks standard deviation 75.8, vs. 4.3 weeks standard deviation 4.9). Unfortunately in Uganda, data collected on the guardian delay and health system delay were only available for those guardians with 3 or less health encounters prior to UCI. The questionnaire was designed to capture guardians’ responses to a series of questions for each health encounter. In Uganda, there was systematic error in data collection that resulted in, after the fourth encounter, failure to document guardians’ responses to the question that assessed time between health encounters. Other questions were unaffected. Among this subgroup of guardians with 3 or less health encounters, total delay was slightly less than the overall Ugandan cohort (median 10.7 weeks, mean 22.0 standard deviation 34.0). Analysis of the components of delay among this subgroup showed that guardian delay also constituted the majority of the total delay (guardian delay median 4.3 weeks, range 0.7-149.9, vs. health system delay median 2.6 weeks, range 0.1-16.0; n = 26). Mean guardian delay was 16.9 weeks (standard deviation 34.1), while mean health system delay was 5.1 weeks (standard deviation 5.0).

### Care-seeking history

Guardians in Kenya most commonly visited dispensaries when initially seeking care for their child (50% of respondents). The next most common facilities for first node of entry were community clinics, mobile clinics, and mission hospitals, each reported by 10%. The remainder of Kenyan guardians first visited a dental clinic, health center (any level), district hospital or JTRH (5% each). In Uganda, 48% of all guardians first sought help at private clinics/hospitals, followed by health centers (any level, 21%), and district hospitals and herbalists (8% each). Seven percent visited a regional hospital and 5% a dental clinic. Out of a total of 82 children with BL, only 8 were correctly diagnosed with a tumor at the initial entry node of care, 7 (11%) in Uganda and 1 (5%) in Kenya. The remainder of children were either misdiagnosed or not informed of a diagnosis at this first encounter.

The majority of guardians in both Kenya and Uganda visited multiple nodes of care before reaching JTRH or UCI, with guardians in Uganda accessing a greater number on average than those in Kenya. In Uganda, 20% of the guardians reached UCI at the 3rd node of care, 28% at the 4th node, 33% at the 5th node, and 20% at the 6th node of care, or thereafter (median 5th node of care, range 3–16). In contrast, 15% of guardians in Kenya reached the care endpoint, JTRH, at the 2nd node of care, 50% at the 3rd node, 30% at the 4th node, and 5% at the 6th node of care (median 3rd node of care, range 2–6). A key distinction between our catchment sites is that UCI serves as a national referral hospital while JTRH is a regional referral center. The geographic areas served by the respective hospitals were roughly equal in size as Kenya is almost twice the size of Uganda, however this could have contributed to the greater number of referrals in Uganda compared to Kenya.

Most common misdiagnoses across all nodes of care in both settings were dental problem/cyst, splenomegaly/abdominal swelling, malaria and non-malarial infection. In Uganda, dental problem/cyst was the most frequent misdiagnosis (observed in 19 encounters), followed by non-malaria infection (n = 11), splenomegaly/abdominal swelling (n = 7) and malaria (n = 7). In Kenya, most common misdiagnoses were splenomegaly/abdominal swelling (n = 10) and malaria (n = 8). Data on treatment were available for only the first three health encounters in both settings. We examined most common treatments prescribed for those encounters that did not yield a tumor diagnosis—a grouping that includes misdiagnoses and encounters in which guardians were not informed of a diagnosis. Out of a total of 119 encounters meeting this criteria in Uganda, guardians most frequently reported receiving unspecified injections/tablets/ointments—as documented in 70 encounters (59%), followed by no treatment—23 encounters (19%). Of note, 16 encounters (13%) resulted in tooth extractions in this setting. Other common treatments reported include: pain medication/analgesics (n = 12, 10%), antibiotics (n = 10, 8%), and eye drops/ointment (n = 5, 4%). This is in contrast to Kenya, where slightly different treatment practices were observed. Of the 37 health encounters in this setting, guardians were most likely to receive no treatment—as reported in 12 cases (32%), followed by anti-malarials (n = 9, 24%), pain medication (n = 6, 16%) and antibiotics (n = 5, 14%). No Kenyan guardians reported tooth extractions prior to arriving at JTRH.

Source of referrals to JTRH and UCI are presented in Table 2. Forty-percent of all guardians learned about JTRH from health centers (any level), with the remainder referred from: dispensaries (15%), dental clinics (15%), friends/neighbors/self (15%), and private/mission hospitals (10%). In Uganda, almost all guardians (81%) arrived at UCI after referral from Mulago Hospital, the Uganda National Referral Hospital located on the same campus as UCI. Given the proximity and affiliation of these centers, we considered referral sources as the last node of care prior to Mulago Hospital or UCI for those referred directly from lower-level facilities. Most common referral sources were district hospitals (32%), regional hospitals (24%), private/mission hospitals (24%) and health centers (any level, 8%).

**Table 2:** Most frequent sources of referrals to Jaramogi Oginga Odinga Teaching and Referral Hospital (JTRH) and Uganda Cancer Institute (UCI) for children presenting with Burkitt lymphoma, 2010

	**Western Kenya (n = 20)**	**Uganda (n = 61) **^**a, b**^
NPGH/UCI referral source:		
Regional Hospital	0 (0)	15 (24.2)
District Hospital	0 (0)	20 (32.3)
Health Center (any)	8 (40.0)	5 (8.0)
Private/Mission	2 (10.0)	15 (24.2)
Dispensary	3 (15.0)	0 (0)
Dental Clinic	3 (15.0)	3 (4.8)
Friend/Neighbor/Self	3 (15.0)	1 (1.6)
Traditional healer/herbalist	0 (0)	2 (3.2)
N/A	1 (5.0) ^c^	0 (0)

^a^N = 61; 1 missing source of referral.

^b^For the purposes of this analysis, UCI and Mulago was considered a single node of care.

^c^1 patient diagnosed during routine care visit.

### Structural factors influencing access to care

Nearly all guardians in Kenya and Uganda relied on public transportation to reach the cancer referral centers, as shown in Table 3. Reported transportation costs to UCI and JTRH were considerable, as was travel time; both of which were found to be slightly higher in Uganda than Kenya. The median cost of transport in Uganda was USD $4.47 (range 0.45-89.44) and the median time to reach UCI was 4 hours (range 0.5-27 hours). In Kenya, guardians paid a median of $3.41 (range 0.37-8.69) and traveled for 2.5 hours (range 0.3-5).

**Table 3:** Structural factors influencing access to cancer care for children with Burkitt lymphoma at Jaramogi Oginga Odinga Teaching and Referral Hospital (JTRH) and Uganda Cancer Institute (UCI), 2010

	**Western Kenya, n (%)**	**Uganda, n (%)**
Region	Nyanza -18 (90)	Central – 27 (43.6)
	Western – 2 [10]	Eastern – 26 (41.9)
		North East – 1 (1.6)
		Southern – 1 (1.6)
		Western – 7 (11.3)
Travel time to UCI/JTRH (hrs)		
Mean (SD)	3.0 (0.3)	5.8 (0.8)
Median (range)	2.5 (0.3-5)	4 (0.5-27)
Transport to UCI/JTRH		
Public transport	19 (95.0)	60 (96.8)
Personal/friend’s transportation	1 (5.0)	1 (1.6)
Mission or NGO vehicle	0 (0)	1 (1.6)
Travel cost to UCI/JTRH from home (USD)		
Mean (SD)	3.91 (0.6)	6.68 (1.43)
Median (range)	3.41 (0.37-8.69)	4.47 (0.45-89.44)
Anyone else available to bring child to hospital (at admission):		
No	7 (35)	32 (51.6)
Yes	13 (65)	29 (46.8)
Support; caring for other young children		
Adult family member	13 (65.0)	46 (74.2)
Neighbor/friend in my home area	0 (0)	1 (1.6)
Older children	4 (20.0)	14 (22.6)
No young children in need of care	3 (15.0)	0 (0)
Perceived barriers to bringing child to the hospital^a,b^		
Money	14 (70.0)	55 (88.7)
Household responsibilities^c^	6 (30.0)	13 (21.0)
Caretaker ill	0 (0)	2 (3.2)
Health system: communication issues, delay in diagnosis or transfer	1 (5.0)	10 (16.1)
Other	2 (10.0)	2 (3.2)
None	2 (10.0)	0 (0)
What would make coming to the hospital easier^b,d^		
Money	12 (60.0)	55 (83.3)
Transport	11 (55.0)	0 (0)
Health system: improved communication, earlier diagnosis	0 (0)	6 (9.1)
Other	4 (20.0)	1 (1.5)

^a^Kenya, 4 missing; Uganda, 3 missing.

^b^More than one applicable; percentages add to greater than 100%.

^c^Includes care of children, family members, and other non-specified household responsibilities.

^d^Kenya, 2 missing; Uganda, 7 missing.

Guardians accompanying the children with BL were all family members and most were the primary caretakers. Many perceived household obligations, including care of their other children, as a major obstacle to seeking care for their ill child. A considerable proportion indicated that no one else was able to bring the child to the hospital (Kenya 35%, Uganda 52%); however, most received some level of instrumental support from family members. About three-quarters in Kenya and Uganda relied on adult family members to care for other children at home; another quarter relied on older children in the household. Notably, only one guardian in either setting received support in the form of childcare from the community.

### Knowledge and concepts concerning disease

Knowledge of childhood cancer in general and BL in particular was limited among participants at JTRH and UCI (Table 4). None of the guardians in Kenya had ever known a child with cancer and few (16%) in Uganda had. Only a small minority - 5% and 19% in Kenya and Uganda, respectively were aware that children could get cancer and even fewer had ever heard of BL (5% both sites). When questioned more broadly on general knowledge and perceptions of cancer, most were uncertain if it was curable (Kenya 65%, Uganda 53%). About 20% of all guardians in Uganda agreed, "cancer is contagious," with 67% undecided on the matter. In contrast, in Kenya, nearly all (90%) disagreed or strongly disagreed with the statement. Stigma associated with cancer was more commonly reported in Uganda than Kenya. Sixty-percent in Uganda felt cancer was stigmatized in their communities; only 20% of guardians in Kenya felt similarly. Overall, guardians in this study did not perceive cancer as a curse or a bewitchment, with most disagreeing when asked directly, as shown in Table 4.

**Table 4:** Guardian’s knowledge and perceptions of cancer, childhood cancer, and Burkitt lymphoma

	**Kenya, n (%)**	**Uganda, n (%)**
Cancer is contagious		
Strongly agree	0 (0)	2 (3.2)
Agree	1 (5.0)	10 (16.1)
Undecided	1 (5.0)	39 (62.9)
Disagree	17 (85.0)	11 (17.7)
Strongly disagree	1 (5.0)	0 (0)
Cancer is a curse^a^		
Strongly agree	0 (0)	1 (1.6)
Agree	1 (5.0)	6 (9.7)
Undecided	1 (5.0)	23 (37.1)
Disagree	17 (89.5)	32 (51.6)
Strongly disagree	0 (0)	0 (0)
Cancer is a bewitchment^b^		
Strongly agree	0 (0)	0 (0)
Agree	1 (5.0)	2 (3.2)
Undecided	0 (0)	17 (27.4)
Disagree	19 (95.0)	43 (69.4)
Strongly disagree	0 (0)	0 (0)
Cancer is curable		
Strongly agree	0 (0)	2 (3.2)
Agree	7 (35.0)	22 (35.5)
Undecided	13 (65.0)	33 (53.2)
Disagree	0 (0)	5 (8.1)
Strongly disagree	0 (0)	0 (0)
Did you know that children can get cancer?		
No	19 (95.0)	48 (77.4)
Yes	1 (5.0)	12 (19.4)
Have you ever known a child who has had cancer?		
No	20 (100.0)	45 (72.6)
Yes	0 (0)	10 (9.7)
Have you ever heard of BL?		
No	19 (95.0)	56 (90.3)
Yes	1 (5.0)	3 (4.8)

^a^Defined as a result of you doing something wrong.

^b^Defined as a result of someone doing a wrong to you.

### Barriers to care: Guardians’ perceptions

The majority of guardians in this study identified financial constraints as the major impediment to bringing their child to the hospital (Kenya 70%, Uganda 89%). In some cases this was described in terms of cost of transportation and others as cost of care (i.e. user fees, diagnostics and treatment). Consistent with these responses, the leading response given for what would make coming to the hospital easier was money. In Kenya, many also indicated provision of transportation, which most likely relates to financial costs as nearly all guardians in this setting—as well as Uganda—relied on public transportation. Household responsibilities, as discussed above, were the second most common response given as a barrier to care after costs, as reported by approximately a quarter of participants in both Kenya and Uganda. For some this was described as responsibilities related to caring for other children or ill family members, and others as unspecified household duties.

### Factors associated with delay: univariate analysis

In this study, we sought to identify independent predictors of total delay, guardian delay and health system delay (Table 5). Factors that were examined include guardian sociodemographic characteristics and knowledge of and attitudes toward cancer, structural factors of healthcare access, symptoms at illness onset and first node of care. Among Kenyan guardians, the following factors were found to be associated with delay: source of income, region and the presence of bleeding gums or loose or painful teeth at onset of illness. Both total delay and health system delay were significantly greater among guardians who identified as self-employed or small-scale business owners as compared to those who were peasant farmers and other occupations (Kruskal-Wallis, p = 0.01 and 0.05, respectively). The sample size was too small to draw any conclusions from comparison from findings related to region and initial symptoms. All but two guardians were from the Nyanza region and only two children initially experienced bleeding gums or loose or painful teeth. None of the other factors investigated emerged as predictors of delay—guardian delay, health system delay, total delay—in this setting.

**Table 5:** Univariate analysis of association between sociodemographic and health-system variables and diagnosis delay among children with Burkitt Lymphoma in Jaramogi Oginga Odinga Teaching and Referral Hospital (JTRH) and Uganda Cancer Institute (UCI), 2010

	**Kenya**						**Uganda**						**Pooled – Uganda and Kenya**	
	**Guardian delay**^**a**^		**Health system delay**^**b**^		**Total delay**^**c**^		**Guardian delay**^**a,d**^		**Health system delay**^**b,d**^		**Total delay**^**c,e**^		**Total delay**^**c,f**^	
	**Mean**	**P**	**Mean**	**P**	**Mean**	**P**	**Mean**	**P**	**Mean**	**P**	**Mean**	**P**	**Mean**	**P**
**Overall**	34.0	--	4.3	--	38.3	--	16.9	--	5.1	--	20.4	--	25.0	--
Guardian														
Mother	38.1	0.58	4.7	0.28	42.8	0.62	17.7	0.62	4.3	0.59	22.4	0.28	29.7	0.74
Father	7.8		1.4		9.1		4.6		7.1		17.4		16.2	
Grandparent	-		-		-		16.3		4.0		16.4		16.4	
Aunt	10.1		2.0		12.1		96.5		6.3		45.7		37.3	
Sibling	44.1		7.3		51.4		2.0		2.3		3.9		15.8	
Self	-		-		-		4.2		13.0		17.1		17.1	
Age (years)														
<30	44.9	0.61	3.1	0.82	48.1	0.70	22.8	0.25	2.9	0.63	16.7	0.20	26.5	0.29
30 - 39	43.9		4.5		48.3		3.9		5.3		18.7		29.4	
>39 - 49	8.8		5.7		14.5		9.3		7.1		17.5		16.7	
>49	16.3		2.0		18.3		29.6		4.7		26.5		26.1	
Primary														
Caretaker														
Yes	34.7	0.21	4.3	0.53	39.0	0.31	18.1	0.34	5.4	0.34	21.8	**0.02**	26.1	0.13
No	27.1		4.6		31.8		2.6		1.6		5.5		13.0	
Education														
Incomplete primary	8.6	0.65	5.7	0.80	14.3	0.76	16.5	0.06	5.1	0.11	20.7	0.12	19.5	0.25
Complete primary	87.9		3.1		91.0		22.0		0.8		24.1		52.8	
Attended secondary	9.8		4.4		14.3		1.2		6.1		10.5		11.0	
Completed secondary	14.7		3.6		18.3		34.5		9.8		40.7		28.3	
Occupation														
Peasant farmer	7.1	0.11	2.1	**0.01**	9.2	**0.05**	17.2	0.06	4.9	0.99	21.0	0.33	18.6	0.09
Self employed/business	66.2		9.1		75.3		49.5		4.1		28.1		48.4	
Other	52.9		2.6		55.5		8.1		5.9		13.5		24.0	
Number of children														
0-2	55.7	0.74	2.6	0.47	58.3	0.79	4.6	0.26	2.4	0.25	9.2	**0.03**	21.5	0.11
3-5	34.6		3.5		38.1		12.4		7.2		20.7		26.0	
≥ 6	10.2		8.6		18.9		33.9		4.7		26.9		25.5	
Care node of first entry														
Regional Hospital	298.0	0.50	10.6	0.76	308.6	0.53	3.0	0.28	1.1	0.06	18.1	0.69	76.2	0.79
District hospital	9.4		2.6		12.0		85.7		4.3		46.6		39.7	
Health center (any)	4.4		5.3		9.6		4.9		8.0		18.4		17.8	
Private/Mission	15.4		11.6		27.0		14.0		4.0		16.9		18.2	
Dental Clinic	199.4		2.0		201.4		3.0		15.7		13.9		60.8	
Mobile Clinic	10.0		3.2		13.2		-		-		-		13.2	
Community Clinic	9.4		3.4		12.9		-		-		-		12.9	
Dispensary	9.9		2.9		12.8		-		-		-		12.8	
Other	-		-		-		10.9		2.0		27.6		27.6	
Care Nodes (#)														
0-2	13.1	0.98	10.2	0.69	23.3	0.93	-	N/A	-	N/A	-	0.15	23.3	0.29
3-4	39.4		3.3		42.8		16.9		5.1		22.6		29.9	
5-6	9.4		2.6		12.0		-		-		15.9		15.7	
>6	-		-				-		-		29.3		29.3	
Referral Source														
Regional Hospital	-	0.82	-	0.89	-	0.97	18.8	0.60	5.1	0.53	21.1	0.74	21.1	0.98
District Hospital	13.4		3.1		16.5		8.1		9.1		20.4		17.8	
Health Center (any)	8.9		2.0		10.9		-		-		-		10.9	
Private/Mission	71.1		3.3		74.4		1.4		2.9		16.3		38.0	
Dental Clinic	-		-		-		7.6		1.0		8.6		8.6	
Friend/Neighbor/Self	15.4		11.0		26.4		-		-		-		26.4	
Other	62.1		5.1		67.3		-		-		-		67.3	
Travel time (hours)														
0 - 2	37.3	0.66	5.1	0.65	42.3	0.60	29.6	0.33	2.6	0.19	20.1	0.39	29.3	0.60
>2 - 4	8.3		3.2		11.5		19.7		6.0		28.6		25.1	
>4 - 8	53.0		4.0		57.0		4.9		6.7		13.8		22.4	
>8	-		-		-		15.0		3.6		17.7		17.7	
Transport														
Public Transport	35.3	0.79	4.3	0.54	39.6	0.66	16.9	N/A	5.1	N/A	21.1	0.62	25.8	0.65
Personal/friend’s	9.8		4.4		14.3		-		-		10.7		12.5	
Mission or NGO	-		-		-		-		-		8.6		8.6	
Travel cost														
0 – 2.49	46.2	0.37	5.6	0.59	51.7	0.38	23.6	0.30	3.7	0.26	24.3	0.72	35.1	0.32
2.50 – 5.00	46.3		2.7		48.9		8.4		3.5		19.4		25.8	
5.01 – 7.50	4.9		2.8		7.7		3.3		4.2		15.0		12.9	
>7.50	8.6		8.6		17.1		21.7		7.7		22.4		22.1	

^a^Mean interval between onset of clinical symptoms and first encounter with health care system.

^b^Mean interval between first encounter with health care system and initiation of treatment at UCI/NPGH.

^c^Mean duration of clinical symptoms on presentation at UGI/NPGH.

^d^Data available for those guardians with 4 or less care nodes in total; n = 26.

^e^N = 59, 3 missing.

^f^N = 79 (Kenya, n = 20; Uganda, n = 59), 3 missing.

In Uganda, guardian delay was influenced by beliefs about the curability of cancer, with delay being paradoxically greater among those who believed cancer is curable (Spearman, p = 0.04). Guardians’ perceptions of cancer as a contagious disease, measured by Likert Scale, were shown to be significantly associated with health system delay in this setting; however, we were unable to identify any meaningful trends in delay across the scaled responses (Spearman, p = 0.04). There was a trend of increased total delay with increased numbers of children in the household (Kruskal-Wallis, p = 0.03). Total delay was also significantly greater among guardians who identified as primary caretakers as compared to those who did not (Wilcoxon rank sum, p = 0.02). Notably, other guardian sociodemographic characteristics, structural factors of healthcare access, symptoms at illness onset and location of first health encounter did not influence delay at this site. We also investigated factors associated with delay in a pooled analysis of all guardians from both sites. Interestingly, none of the variables examined showed an independent association.

## Discussion

Our study is one among a small number of studies addressing delays in diagnosis and treatment of childhood cancers in low- and middle-income countries (LMIC). We identified 5 earlier studies, 2 of which focused on leukemia, 1 on retinoblastoma and 2 on all childhood cancers [11-14]. To our knowledge, our study is the first that focuses exclusively on diagnosis delays among children with BL, which is a physically evident disease and therefore easier to detect by untrained parents. In both Kenya and Uganda, we observed long delays from onset of symptoms to first treatment, with the median total delay about 12 weeks in both settings. In comparison, a recent meta-analysis that pooled data on time to diagnosis among Non-Hodgkin Lymphoma patients in HICs (n = 1081) reported a median total delay of 3.8 weeks [15]. Findings from the earlier studies addressing delays in diagnosis in LMIC were varied. Median total delays were 4.1 and 4.4 weeks for leukemia, 15 weeks for retinoblastoma, and 4.9 and 13.1 weeks for studies including all childhood cancers. Of note, Brown et al. reported results on delay in Nigeria stratified by diagnosis and found median delay among children with BL was 9.7 weeks, slightly shorter than present findings [11].

In both of our study populations, guardian delay constituted the majority of the total delay—74% in Kenya and 65% in Uganda. Median health system delay was 2 weeks in Kenya and 2.6 weeks in Uganda, Length of health system delay at both sites was comparable to data from earlier studies on delays for other childhood cancers in LMIC: in Nicaragua (2 weeks) and South Africa (3 weeks), although less than delays observed in Nigeria (9 weeks) [11,13,14]. In contrast, guardian delay in the present study was considerably longer than previous reports—9 weeks in Kenya and 4.6 weeks in Uganda as compared to 1 to 2 weeks in other LMIC settings studied [11,13,14]. Overall, our findings differ from those previously reported, which have shown a tendency for guardian delay to be less than health system delay, both in LMIC and HIC [11,13,14,16].

The long guardian delay in our study may reflect guardians’ perceptions of illness, misinterpretation of early symptoms, poor access to health care facilities (distance and transportation availability and financial constraints) and competing household and work responsibilities. Although delays in diagnosis appear to be largely due to guardian delay in our study population, we also observed considerable variation in delay among respondents. That is, health system delay emerged as the main component of total delay for many—15% in Kenya and 42% in Uganda. Across both settings, guardians reported multiple care nodes and complex and indirect pathways of navigation. Prolonged health system delay in these cases and others may be partially explained by limited knowledge of BL among health service providers, insufficient clinical investigations or failure to refer patients to heath facilities with resources for further management. Another important consideration is the limitations of the guardian delay versus health system delay dichotomy. This construct establishes two separate time intervals to attempt to isolate and examine delay that may be explained by guardian-level factors separately from that which may be attributable to the health system. This framework relies on the false assumption that there is no interplay between these factors. That is, it implies that after a child reaches the first health facility, the care-seeking experience is essentially sheltered from guardian-level influences like region, cost and availability of transportation and health beliefs. The limitations of this construct become most apparent in the context of multiple visits to care facilities as many of the same factors influencing guardian delay may contribute to delays in pursuing follow up care. Additional studies would be necessary to disentangle the longitudinal contribution of these elements.

Few of the factors investigated were found to be associated with guardian delay or health system delay in Kenya and Uganda. Many of the guardian socio-demographic characteristics did not influence delays in either settings, including age, education, occupation and select indicators of socioeconomic status (housing, windows, home cooking apparatus, water source and animals in home). Past research that has examined the influence of education on diagnosis delay in childhood cancers has been inconsistent. Several studies in LMIC and HIC have shown shorter total delay [16,17] and guardian delay [12,18,19] with increased levels of education, yet other studies—conducted in Nigeria and South Africa—have found education to have no significant effect [11,14]. With few respondents reporting education beyond primary school, it is possible our study population was too homogenous to discern any significant association.

In Kenya, we found guardian’s relationship to the child had no effect on delay. However, in Uganda, total delay was found to be greater among those who identified as primary caretaker as compared to those who did not. These findings may reflect the influence of several factors on primary caretakers and their access to care, including household and work obligations and the availability of social support. The benefit of social resources, from family and/or friends, in overcoming barriers to healthcare utilization has been well characterized throughout the literature [20]. In the present study, support largely came from other family members rather than the community, with nearly all guardians reporting shifting roles among families as a consequence of the child’s illness.

Interestingly, there was no association between select indicators of socioeconomic status and delay in either Kenya or Uganda. Structural factors including travel time and costs of transportation to the JTRH and UCI also did not emerge as independent predictors of delay in our analysis. Our results contradict previous studies in Kenya and Uganda, which have shown reduced access to and utilization of health services among lower socioeconomic groups, with distance to the facility and costs of care among the key barriers [20-23]. These earlier findings are supported in our study, however, by guardians’ perceptions of the barriers to care. When asked what was most difficult about bringing the child to the hospital, financial issues were the leading concern, often related to costs of transportation and clinical care. Many of the guardians traveled long distances to reach JTRH and UCI and nearly all used public transportation. Travel time and transportation costs were particularly high in Uganda, where UCI serves a broader catchment area as the national cancer referral center; JTRH in contrast is one of several regional cancer referral centers in Kenya. In both of these settings, guardians reported multiple health encounters prior to JTRH and UCI, resulting in accumulating costs, of travel, user fees, diagnostic tests and treatment, none of which were assessed in the present study. Thus, it is conceivable that measured costs were too narrow in scope to observe any correlation. Future studies should explore the impact of these additional costs on delay, as well as indirect costs such as loss of daily income.

Our results related to guardians’ knowledge of cancer indicate low awareness of cancer among the general community. Interestingly, however, knowledge of cancer or specifically BL did not appear to influence delay in either setting. This suggests interventions aimed solely at increasing disease awareness may be insufficient for improving early diagnosis. Also evident from these findings is that guardians arrive at UCI and JTRH with a limited understanding of their child’s illness despite multiple encounters within the health care delivery system leading up to admission to the cancer center. This may partially reflect the fact that many children remained undiagnosed or were misdiagnosed at lower-level facilities; however, insufficient communication between health providers and guardians may also be a contributing factor.

Perceptions of cancer were largely similar among guardians in Kenya and Uganda. Few thought cancer was contagious or viewed the illness as a curse or a bewitchment. One exception was stigma, which was more common in Uganda than Kenya. Although past studies have shown stigma and fears of discrimination can be an important barriers to care, these sentiments were not associated with delay in the present study [24]. Overall, few guardians in Uganda and Kenya perceived cancer as curable. In Uganda, surprisingly, guardian delay was significantly greater among those who did. We believe this may be a spurious association, however, as few of these guardians (17%; 4 of 24) suspected their child’s illness was cancer before seeking care. In both settings, misconceptions related to the curability of BL have important implications for efforts to improve early diagnosis. Guardians who learn or suspect their child has cancer may be less likely to utilize health services if they perceive treatment will have little to no benefit [25]. Understanding that BL is a curable illness provides hope and reason to pursue care.

Earlier research has shown that initial presentation of symptoms can influence time to diagnosis for other malignancies, including retinoblastoma, brain tumors and leukemia [12,16,17,26]. In the present study, we found no difference in delay according to early symptoms or guardians’ interpretations of the symptoms. These findings may be attributable to the non-specific symptoms observed in early BL. This non-specific presentation, and BL’s relative rarity, underscores the inherent challenges of timely diagnosis of this disease. Symptoms common to many conditions can lead not only to varying perceptions of illness among guardians, but also frequent misdiagnosis in clinical settings. An example in malaria holoendemic areas where splenomegaly is common would be the misdiagnosis of an abdominal tumor located on the left side as opposed to the right side of the body.

Guardians in both settings experienced lengthy health-seeking itineraries, often consisting of several different types of health facilities. Guardians most commonly first sought care in Uganda at private clinics, and in Kenya, at dispensaries; about half visited other care providers. Notably, almost all guardians reported that they first sought western medicine and not care from traditional healers. It is possible however that use of alternative forms of care such as traditional medicine may be underreported due to perceived stigma. Although various levels of health facilities typically differ in their human resources and institutional capacities, there were no significant differences in delay according to the first point of care. Brown et al. reported similar results related to total delay, but found increased guardian delay at tertiary care centers when compared to lower-level facilities [11]. Many of the health encounters in our study resulted in sub-optimal care, including misdiagnoses and inappropriate treatment, particularly at lower-level facilities. We examined treatment practices across various levels of the health system however data were insufficient to identify any trends. Future studies should explore this relationship as any findings may yield valuable information for improving care delivery. Overall, our results suggest that interventions designed to address diagnosis delays should include outreach and continuing education for health professionals across multiple levels of the health system. Local providers must be able to recognize early signs of cancer and make appropriate referrals and adequately communicate the need for haste to the parents.

An earlier study on guardians’ perspectives of barriers to pediatric cancer care was conducted at UCI in 2007, three years prior to our study (K. Stiffler, University of Washington School of Public Health Masters thesis). A similar survey was administered to 32 guardians of children with cancer on arrival to UCI; 23 of the children were diagnosed with BL. Comparison of findings from the two studies yields interesting insight into possible changes in health-seeking behaviors among guardians in Uganda, and their experiences navigating the health-system for effective treatment.

Overall, the mean total delay, from onset of symptoms to arrival at UCI, remained largely similar from 2007 to 2010 at 18.5 and 20.4 weeks, respectively. In contrast, there was a shift in the main component of total delay, from health system delay in 2007 to guardian delay in 2010. The mean health system delay decreased from 69% of the total delay (12.9 weeks) in 2007 to 23% in 2010, whereas the mean guardian delay increased from 31% of the total delay (5.2 weeks) to 77%. This comparison is limited by several factors, including slight differences in study procedures and questionnaires, and missing 2010 data on guardian delay and health system delay, as discussed previously. With data only available for guardians with 3 or less health encounters prior to UCI, it is possible that 2010 estimates of health system delay represent a slight underestimation. Despite this limitation, the marked difference between these two estimates suggests that the time to UCI after guardians first seek care may be decreasing. One possible explanation may relate to differences in the study population, with guardians from 2010 representing a slightly broader catchment area. Mean travel time among this group was 5.8 hours as compared to 2.9 hours in 2007. This could reflect increasing country-wide awareness of cancer due to the collaboration between UCI and the Fred Hutchinson Cancer Center and the formation of The Uganda Program on Cancer and Infectious Disease that has been receiving a lot of media attention. Both studies identified transportation and financials costs as the most important barriers to seeking care.

Many of the factors investigated in the present study, including knowledge and attitudes toward cancer and structural barriers to care, contribute not only to late stage of presentation, but also refusal and abandonment of therapy, one of the leading causes of poor outcomes in resource-limited settings [27]. For example, in a recent study on BL in Nigeria where parents were required to pay for chemotherapy and laboratory diagnostics, only half of all children presenting to the hospital completed treatment due to financial constraints; 20% never initiated treatment and another 32% abandoned treatment due to costs [19]. Israëls et al. also reported similar findings from a study on abandonment among guardians of children with BL and Wilms tumor at a public hospital in Malawi, where treatment is provided free of charge [28]. Factors influencing abandonment included financial costs related to transportation, food and loss of income; household responsibilities; and perceptions related to the curability of cancer.

Our study has several limitations. The sample sizes in both Kenya and Uganda were relatively small. Secondly, all data were self-reported. Guardians were asked to report on several variables across multiple health encounters that took place over an extended period of time. Thus, recall bias is a concern. Perhaps most importantly, our findings represent only those guardians who successfully navigated the health system, received appropriate referrals and arrived at the cancer centers, UCI and JTRH, for treatment. Many other children with BL in both settings may have been less fortunate and remained undiagnosed or mismanaged at lower-level facilities. Community-based investigations of the health-seeking experiences would provide additional insight into the barriers to diagnosis and treatment, including factors that may have not been examined in the present tertiary care, hospital-based setting.

Using guardian delay and health system delay to analyze time intervals across the care-seeking experience also has several limitations. One source of bias stems from our methods of measuring guardian delay, which relied on questions of when symptoms were first recognized and when care was first sought. Assessing symptom recognition and interpretation presents many difficulties that have been well characterized throughout the literature [29]. Although this discussion is beyond the scope of this paper, it is important to note that our analysis relied on the flawed assumption that all guardians recognize and interpret symptoms in similar ways. A more comprehensive inquiry into guardians’ perceptions of symptoms would provide a more accurate and precise assessment of guardian delay. Lastly, many of the same factors influencing guardian delay can influence health system delay, particularly with multiple health encounters, as previously discussed.

## Conclusions

This study aimed to examine delays in the diagnosis and treatment of children with BL in Uganda and western Kenya, and to identify the factors that may contribute to these delays. We found that prolonged delays are common in both settings, with much of the delay occurring prior to the first health encounter. Consistent with earlier studies of other childhood cancers, we found that no single factor emerged as a major cause of delay, but rather a confluence of factors was implicated [11-14,16,18,30]. Among guardians, structural barriers emerged as the leading concern, particularly those related to costs of transportation and clinical care. Searching for an accurate diagnosis and effective treatment was a resource-intensive and complex endeavor for many guardians, requiring multiple visits to different health facilities and care providers. During these visits, many children remained undiagnosed or were inappropriately treated before reaching the cancer referral centers.

Our findings related to mismanagement of BL cases at lower level facilities highlights an important area of weakness in the health system contributing to late stage of presentation and poor outcomes among children with BL and likely childhood cancers in general. In both Uganda and western Kenya, recent efforts have been made to shift cancer care modalities to lower level facilities to improve access to care. We believe that our findings support the argument that the best allocation of resources involves continued development of strong centralized cancer centers with efficient referral systems. This notion is support by a recent review of best-practice care models for national cancer plans [31].

Overall, our study suggests that multidimensional interventions are likely required to address late stage of presentation among children diagnosed with BL. Based on our findings, we recommend that efforts aimed at reducing delays in diagnosis consider the following actions; 1) Targeted health education of guardians on the importance of seeking early care for children’s illnesses; 2) Increase awareness of childhood cancers among the general public using effective means of communication; 3) Minimize structural barriers to care; 4) Continuing education programs for health providers on signs and symptoms of common childhood cancers, including providers at lower-level facilities; 5) Improve referral linkages between community health providers and cancer referral centers.

## Methods

### Study design

This research study was designed to collect quantitative and qualitative data from guardians of children diagnosed with BL at major cancer referral centers in Uganda and western Kenya through use of standardized questionnaires. The primary objectives of this study are to identify barriers to prompt diagnosis and treatment of children with BL in Uganda and western Kenya and to examine the factors that influence the health-seeking behaviors of guardians whose children have been diagnosed with BL in these settings. The study was carried out at the Jaramogi Oginga Odinga Teaching and Referral Hospital (JTRH), formerly named the New Nyanza Provincial General Hospital, Kisumu, Kenya and Uganda Cancer Institute (UCI), Kampala, Uganda. Enrollment was initiated in January 2010 and continued through December 2010.

### Study site and population

JTRH is a public regional referral hospital, which serves as the referral center for children diagnosed with cancer in western Kenya. It is located in an urban setting in Kisumu, Kenya, the third largest city in the country. UCI is a public referral comprehensive cancer center that is adjacent to Makerere University College of Health Sciences and Mulago hospital; it is the national referral center for childhood cancer in Uganda. Kampala is the largest city in Uganda. Guardians were eligible if their child was diagnosed with BL and admitted to JTRH or UCI for treatment, and they were able and willing to give informed consent. If more than one parent or guardian was present, both were eligible to participate individually. Study participants were excluded if they spoke languages other than those spoken by the study investigators or key personnel.

### Recruitment

Staff at JTRH and UCI identified guardians eligible for recruitment and invited them to participate in a research questionnaire study. Potential study participants were informed of the study details, read the consent form, and provided the opportunity to ask questions, as well as consult with other family members. Interested and willing guardians were administered a short quiz to assess understanding of study procedures. Informed consent was obtained from guardians who demonstrated an adequate understanding—verbal consent in Kenya and written consent in Uganda. Those with quiz performances that suggested they did not understand the study and implications of participation were excluded.

### Data collection tools

We designed standardized questionnaires to be administered to guardians as structured face-to-face interviews during the child’s first or second treatment course. The questionnaires were developed based on the conceptual framework of the Health Belief Model [25] using information gathered through semi-structured interviews of 8 key informants involved in the care of children with BL at JTRH and UCI, which were conducted in June and July 2008. Key informants included physicians, nurses, and other supportive staff. During these interviews, we sought to identify reasons for delayed diagnosis and treatment of children with BL in Uganda and western Kenya. The questionnaires were then beta-tested at JTRH and UCI over a four-week period with guardians of children who were diagnosed with BL and receiving care as in-patients at the time. Questionnaires were then further refined and prompts written after each question to classify type of response if ambiguity unavoidably remained in translation.

The questionnaire collected information on the following factors: demographics (e.g. home location, occupation, schooling, religion, etc.), barriers to health care utilization identified by the guardians (ex. distance to health care center, cost of transport, inability to leave other children, etc.), series of events leading up to the child’s arrival at the study site including each provider and place where care was sought and what diagnosis and treatment were given. The questionnaire also assessed knowledge, attitudes and health-seeking behaviors of the parent (e.g. use of traditional healers or traditional medicines). Pertaining to attitudes, we investigated beliefs about the curability of cancer and perceptions of cancer as a curse or a bewitchment. We also assessed conceptions of stigma associated with cancer in the community using local terms for stigma and related feelings of guilt and shame.

In Uganda, the questionnaires were translated from English into Lugandan, the most commonly spoken language in Uganda. In Kenya, the questionnaires were translated from English into Kiswahili (the second national language) as well as into Dholuo (the language of the Luo tribe, the most populous tribe in Nyanza province). Questionnaires and consent forms were back-translated into English at both sites to verify the accuracy of the translations.

### Data collection

Once participants were recruited and gave informed consent, a trained member of study personnel administered the questionnaire during the child’s first or second treatment course by means of face-to-face interview as described above. The questionnaire was read to all participants in their preferred language irrespective of literacy, and responses were recorded on the questionnaire form. Interpreters were utilized for those guardians preferring languages other than English or Lugandan (in Uganda), and English, Kiswahili or Dholuo (in Kenya).

### Definitions

Prior studies on delays in childhood cancer care have utilized constructs to analyze time intervals across the care-seeking experience. One common approach divides the time lapsed for the entire care-seeking experience, also referred to as "total delay," into two subcomponents, "guardian delay" and "health system delay" [14,16]. Total delay, in the present study, is defined as the time interval from the onset of disease-related symptoms to arrival at JTRH /UCI for treatment; guardian delay, the time period from recognition of symptoms to first encounter with the healthcare system; and health system delay, the time period from the first encounter with the health system to arrival at JTRH /UCI for treatment. "Care node," a term originally introduced by Dye, et al., refers to sites delivering health services, including all levels of facilities and providers (ie. tertiary care centers, regional hospitals, health centers, traditional healers/herbalists, etc.) [32].

### Data processing and analysis

Data were entered into Microsoft Excel, reviewed for consistency and completeness, and analyzed with the use of STATA statistical software package 11.0 [33]. Categorical variables were described with frequency distributions, and continuous variables, with parametric tests. Comparisons of categorical variables between the two sites were calculated with Chi-square test or Fisher’s exact test when needed. Normality of continuous data was assessed using the skewness-kurtosis test, which found the following to be skewed: total delay, guardian delay, health system delay, total care nodes, travel time to JTRH /UCI and transport costs. Thus, comparisons related to these variables were computed by non-parametric methods: The Wilcoxon rank sum test and the Kruskal-Wallis test were employed for comparisons of delay by different categorical variables among each site. Analyses of delay across ordinal variables were carried out with the Spearman correlation. For all tests, p < 0.05 was considered to be statistically significant.

### Ethical considerations

The institutional ethical review committees of the University of Massachusetts Medical School, the University of Washington, Kenya Medical Research Institute, Makerere University, Mulago Hospital, and the Uganda National Council for Science and Technology reviewed and approved all study procedures. All study personnel were trained in ethical issues related to human subjects or were certified by the CITI or NIH Web tutorial. Study participants were informed about the study objectives and procedures for data collection, and their right to refuse to participate, to decline to answer any questions, and to withdraw from the study at any time. Verbal or written consent was obtained from all study participants. All interviews were carried out by trained local study personnel who were fluent in the local languages spoken in their respective countries.

## Abbreviations

AIDS: Acquired immunodeficiency syndrome; BL: Burkitt lymphoma; HIC: High-income countries; HIV: Human immunodeficiency virus; IQR: Interquartile range; JTRH: Jaramogi Oginga Odinga Teaching and Referral Hospital; KEMRI: Kenya Medical Research Institute; LMIC: Low- and middle-income countries; SD: Standard deviation; UCI: Uganda Cancer Institute; USD: US dollars.

## Competing interests

The authors declare that they have no competing interests.

## Authors’ contributions

GCB performed the data analysis, interpreted results and drafted the manuscript. JPC made substantial contributions to the conception and design of the study, was involved in data acquisition, analysis, interpretation and drafting the manuscript. POS made substantial contributions to the conception and design of the study, interpretation of the data and reviewing the manuscript. BN made substantial contributions to the conception and design of the study, acquisition and interpretation of the data and reviewing the manuscript. DO made substantial contributions to the conception and design of the study, acquisition and interpretation of the data and drafting the manuscript. KS made substantial contributions to the conception and design of the study, acquisition, analysis and interpretation of the data and drafting the manuscript. CC made substantial contributions to the conception and design of the study, interpretation of the data and reviewing the manuscript. JAO made substantial contributions to the conception and design of the study, interpretation of the data and reviewing the manuscript. JO made substantial contributions to the conception and design of the study, interpretation of the data and reviewing the manuscript. AMM made substantial contributions to the conception and design of the study, analysis and interpretation of the data and reviewing the manuscript. All authors read and approved the final manuscript.
